# Dynamics of Cell Death After Conventional IRE and H-FIRE Treatments

**DOI:** 10.1007/s10439-020-02462-8

**Published:** 2020-02-05

**Authors:** Borja Mercadal, Natalie Beitel-White, Kenneth N. Aycock, Quim Castellví, Rafael V. Davalos, Antoni Ivorra

**Affiliations:** 1 Department of Information and Communication Technologies, Universitat Pompeu Fabra, Roc Boronat, 138, 08018 Barcelona, Spain; 2 Department of Biomedical Engineering and Mechanics, Virginia Polytechnic Institute and State University, 325 Stranger St, Blacksburg, VA 24061 USA; 3 Serra Húnter Fellow Programme, Universitat Pompeu Fabra, Barcelona, Spain

**Keywords:** Irreversible electroporation, High-frequency irreversible electroporation, Membrane permeability, Caspase 3/7, Bipolar pulses

## Abstract

High-frequency irreversible electroporation (H-FIRE) has emerged as an alternative to conventional irreversible electroporation (IRE) to overcome the issues associated with neuromuscular electrical stimulation that appear in IRE treatments. In H-FIRE, the monopolar pulses typically used in IRE are replaced with bursts of short bipolar pulses. Currently, very little is known regarding how the use of a different waveform affects the cell death dynamics and mechanisms. In this study, human pancreatic adenocarcinoma cells were treated with a typical IRE protocol and various H-FIRE schemes with the same energized time. Cell viability, membrane integrity and Caspase 3/7 activity were assessed at different times after the treatment. In both treatments, we identified two different death dynamics (immediate and delayed) and we quantified the electric field ranges that lead to each of them. While in the typical IRE protocol, the electric field range leading to a delayed cell death is very narrow, this range is wider in H-FIRE and can be increased by reducing the pulse length. Membrane integrity in cells suffering a delayed cell death shows a similar time evolution in all treatments, however, Caspase 3/7 expression was only observed in cells treated with H-FIRE.

## Introduction

Electroporation is a biophysical phenomenon in which cells, when exposed to high electric field magnitudes, exhibit increased membrane permeability to ions and macromolecules. This phenomenon has multiple applications in medicine and biology. Among them, irreversible electroporation (IRE) has proved to be safe and effective for the treatment of solid tumors with a non-thermal mechanism, offering a number of advantages compared to other ablation techniques.^1^,^10^,^36^ IRE treatments rely on the electroporation phenomenon to kill cells by producing nano-scale defects in cellular membranes, which allow for increased transmembrane transport and eventually cause loss of homeostasis. These treatments usually consist of the insertion of metal electrodes directly into the target tissue, and delivery of multiple electric pulses with 70–100 *μ*s pulse length.

The high voltage pulses delivered in IRE treatments can cause electrical stimulation of excitable cells in the body, putting patients at risk for cardiac arrhythmias and leading to undesirable effects such as muscle contractions and acute pain. In order to minimize the risks associated with electrical stimulation, IRE pulses must be synchronized with cardiac activity. In addition, administration of neuromuscular blocking agents and anesthesia are often necessary, increasing the complexity and cost of the entire clinical procedure.

To address these issues, high-frequency IRE (H-FIRE) was proposed as an alternative strategy.^2^ H-FIRE consists of the delivery of bursts of short bipolar pulses (1–5 *μ*s pulse length) instead of the 70–100 *μ*s monopolar pulses typically used in IRE, and has been successfully applied to treat solid tumors with minimal muscle contractions.^29^,^35^,^42^,^43^,^51^ The electric fields required to kill cells with H-FIRE are significantly higher than in conventional IRE protocols,^38^,^39^ but when moving from monopolar IRE waveforms to bipolar H-FIRE bursts, the excitation thresholds of peripheral nerves increase even more significantly than the thresholds for cell death.^26^ Thus, it is possible to minimize muscle contractions while maintaining treatment efficacy with H-FIRE.

Although the ultimate goal of an ablation procedure is to kill all cells within a target volume, the cell death mechanisms are important as they determine the immune response after the treatment.^37^ Cell death can be classified into two main categories, accidental and regulated cell death.^13^ Accidental cell death (ACD) is a virtually instantaneous and uncontrollable form of cell death corresponding to the physical disassembly of the plasma membrane caused by extreme physical, chemical, or mechanical cues. Regulated cell death (RCD) is a form of cell death that results from the activation of one or more signal transduction modules, and hence can be pharmacologically or genetically modulated. In the past, ACD was considered equivalent to necrosis and RCD equivalent to apoptosis. However, in the present, several forms of RCD have been identified besides apoptosis. In addition, some of these non-apoptotic RCD have been shown to act as “programmed necrosis”.^12^,^13^

The exact death mechanisms of conventional IRE treatments are still under study. In the recent past, it was believed that IRE caused a combination of ACD and apoptosis.^9^,^46^ In fact, several studies seemed to support the idea that IRE cell death after the delivery of 100 *μ*s pulses was in part apoptotic.^7^,^20^,^23^,^53^,^54^ However, our understanding of the death mechanisms has quickly evolved in recent years and more recent studies point towards other forms of RCD such as pyroptosis or necroptosis rather than apoptosis.^25^,^55^

Currently, very little is known regarding the cell death dynamics and mechanisms of H-FIRE and how they differ from those of conventional IRE treatments. The pulse lengths that make up H-FIRE bursts have been relatively unexplored^49^ and the cell death dynamics and mechanisms after the delivery of bipolar pulses have been scarcely studied. To the best of our knowledge, cell death dynamics in H-FIRE have only been evaluated by Sano *et al.*^39^ and it was found that both immediate and delayed cell death occurred in cells treated with H-FIRE. The aim of this study is to further investigate these two dynamics in H-FIRE as well as in conventional IRE protocols by quantifying the range of electric fields that lead to each form of death. In addition, we investigate whether the RCD mechanisms leading to delayed cell death are the same in H-FIRE and in conventional IRE with monopolar pulses.

## Methods

3D cell cultures have been previously used to study the outcome of IRE treatments in different cell lines.^3^,^19^,^48^ With this model, it is possible to accurately define the boundary between treated and untreated cells when used in combination with a live/dead staining method. The profile of the treated region can be correlated with the electric field distribution obtained with a numerical model in order to determine the threshold electric fields. Thus, IRE thresholds can be measured with a relatively low number of experiments using this methodology. In the present study, 3D cell cultures were treated with different protocols and the treatment outcomes were measured at two time points (3 and 24 h). This allowed us to identify the regions of immediate and delayed cell death after the treatments and to quantify the electric fields that led to each of them.

Then, in order to gain deeper insight into the dynamics of delayed cell death, cells were treated with a uniform electric field. This series of experiments was performed using cuvettes to treat cell suspensions. First, the electric field thresholds for cell death were measured for IRE and H-FIRE. These electric fields were then delivered to the cells and membrane integrity as well as Caspase 3/7 activity was assessed at different times after treatment.

### Cell Culture

Human pancreatic adenocarcinoma cell line BxPC-3 (American Type Culture Collection, ATCC, Manassas, VA, USA) was grown in RPMI-1640 (ATCC modification) medium (Gibco, Dublin, Ireland) supplemented with 10% fetal bovine serum (Sigma-Aldrich, St. Louis, MO, USA) and 1% penicillin/streptomycin (Invitrogen, Carlsbad, CA, USA). Cells were incubated at 37 °C in a humidified environment containing 5% CO_2_ and subcultured regularly.

### Collagen Scaffold Fabrication

Collagen I hydrogel-based scaffolds were fabricated following the same procedure as in Szot *et al.*^44^ Briefly, concentrated collagen stock solutions were created using rat tail collagen type I. 10% of the total volume of 10× culture media and 2% of the collagen volume of 1N NaOH were added to the collagen solution and mixed thoroughly with a spatula until homogeneous. 1N NaOH was added until the pH was adjusted to 7–7.4 (confirmed visually). Cells in suspension were mixed into the collagen solution for a final concentration of 10^6^ cells/mL. Then, the collagen was injected into custom made polydimethylsiloxane (PDMS) disk-shaped inserts (10 mm diameter and 1 mm height) placed at the bottom of wells in a 24-well plate. The injected collagen was flattened with a PDMS mold and incubated for 20 min to complete polymerization. Finally, the molds were withdrawn, fresh media was added to the wells and they were incubated overnight before performing treatments.

### Electric Field Generation

**Figure 1:**
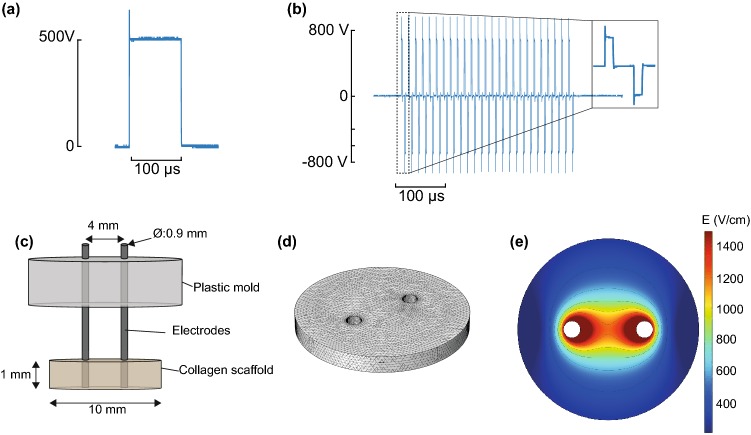
3D cell culture models offer an elegant tool to study lethal electric field thresholds and temporal cell death trends. Oscilloscope traces measured during (a) a conventional IRE treatment (100 *μ*s monopolar) and (b) a 2-5-2 H-FIRE treatment. (c) Schematic of the set-up used to treat 3D cell cultures. (d) FEM mesh used to simulate the electric field distribution in the 3D cell culture. The collagen scaffold was modeled as a disk with a diameter of 10 mm and a thickness of 1 mm. The electrodes were modeled as 0.9 mm diameter cylinders. (e) Electric field distribution in the 3D cell culture with 500 V applied between the electrodes. Note that since the model is linear, these results can be scaled for the different voltages used in the study.

In this study, cells were exposed to a typical IRE protocol (100 *µ*s, monopolar pulses) and H-FIRE protocols with different pulse lengths and inter-pulse delays (See Figs. 1a and 1b). All H-FIRE protocols had an energized time of 100 *μ*s per burst. Throughout the manuscript, H-FIRE protocols will be referred to as positive pulse length—inter-pulse delay—negative pulse length (in *µ*s). All treatment protocols consisted of 80 pulses (or bursts in the case of H-FIRE) delivered at a repetition rate of 1 Hz. Conventional IRE pulses were generated by a BTX ECM 830 pulse generator (Harvard Apparatus, Holliston, MA). H-FIRE bursts were generated by a custom-built pulse generation system (EPULSUS® FBM1-5, Energy Pulse Systems, Lisbon, Portugal).

### Determination of Lesion Areas and Electric Field Thresholds in 3D Cell Cultures

Prior to electroporation treatments, culture media was aspirated from the wells. Electric fields were delivered using blunt tip stainless steel needles with an outer diameter of 0.9 mm. The two electrodes were placed using a custom-made plastic structure which ensured a 4 mm separation between the electrode centers (See Fig. 1c). After treatment, fresh media was added to the wells and they were returned to the incubator. Either 3 or 24 h after treatment, media was aspirated and a PBS solution containing 20 *µ*M Propidium Iodide (PI, Invitrogen) and 2 *µ*M Calcein AM (Invitrogen) was added to the wells. Cells were then kept at room temperature for 30 min protected from light. After washing the cells with PBS (Gibco), images of each well were taken using an inverted DMI 6000B microscope (Leica Microsystems, Wetzlar, Germany) with a 5× objective. In normal conditions, PI is not permeant to cells; however, it can diffuse through the compromised membranes of dead cells. Once inside the cell, this dye binds to DNA and its fluorescence is enhanced 20–30 fold. Calcein AM, in contrast, is a cell permeant dye that emits green fluorescence after internalization and hydrolysis by live cells. This allows for visualization of the distinct regions in the collagen scaffold in which cells are dead or alive after treatment under a fluorescence microscope.

The images obtained were used to measure the lesion area. This was done following the same procedure as Wasson *et al.*^48^ Briefly, the green channel (Calcein AM) was used to define the limits of the lesion area using a custom developed MATLAB code. Once the lesion limits were extracted, the area was calculated by a built-in MATLAB function. In cases where the algorithm was unable to find the lesion limits, the area was manually measured using ImageJ.^41^

Electric field thresholds were calculated by fitting extracted areas (from the images) to results of a numerical model. First, the electric field distribution was computed with a finite element method (FEM) model using COMSOL Multiphysics v4.4 (Stockholm, Sweden) (See Figs. 1c, 1d, and 1e). Areas enclosed by isolines at different electric field values were extracted from the obtained electric field distribution. These values were used to determine a relationship between the electric field and the area by a polynomial adjustment with the least squares method. Finally, the function obtained by the adjustment was used to convert the lesion areas to electric field threshold values. To test the robustness of the whole process, two different voltages were delivered for each treatment protocol and the obtained threshold values were compared statistically.

### Statistical Analysis

A Mann–Whitney–Wilcoxon test was used to analyze differences between lesion sizes and electric field thresholds for each treatment protocol.

Due to the electric field distribution in our experiments, the relation between the lesion size and the threshold electric field is highly non-linear. As a consequence, for a given treatment protocol the relative increase in the lesion size between 3 and 24 h can greatly vary depending on the final lesion size. In other words, unlike the electric field, the lesion size cannot be scaled linearly with the applied voltage and as a consequence the relative increase in lesion size will depend on this voltage. Thus, lesion size measurements were not considered for comparisons between treatment protocols. Instead, to compare the treatment protocols in terms of the proportion of immediate and delayed cell death, a threshold ratio was defined as the mean lethal threshold at 24 h divided by the mean threshold at 3 h. According to this definition, a ratio of 1 would mean that both thresholds are equal and therefore the entire lesion was produced by immediate cell death. In contrast, decreasing values of the ratio would mean that a larger portion of the lesion is produced by a delayed cell death. After calculating the threshold ratios for each treatment protocol, pairwise comparisons between the obtained values were performed using a *t* test. All statistical tests were run using the R-3.5.3 software package.^33^

### Preparation and Treatment of Cells in Suspension

At 70–90% confluence, cells were detached using a solution consisting of PBS and 0.5 mM EDTA (Panreac, Barcelona, Spain). This detachment method was used to facilitate the quick adhesion of cells after treatment. Once detached, cell suspensions were centrifuged twice and cells were resuspended in growth media at a concentration of 5 × 10^6^ cells/mL. 100 µL of suspension was transferred to a 1 mm gap electroporation cuvette (Thermofisher, Waltham, MA, USA) and the desired pulsing protocol was delivered. Immediately after treatment, cells were diluted tenfold in growth media, seeded into 6 well plates and kept in the incubator until further measurements.

### Electric Field Thresholds in Suspended Cells

Twenty-four hours after treatment, cell viability was assessed through a Trypan Blue exclusion assay. Cells were trypsinized and the cell suspension was mixed with a 0.4% Trypan Blue solution (Gibco). Live cells were counted with a Neubauer chamber under a light microscope and viability was calculated as the number of viable cells divided by the number of viable cells in the Sham groups. The threshold was defined as the lowest electric field that resulted in mean viability levels lower than 10%.

### Membrane Permeability to Yo-Pro-1 and Propidium Iodide

Treated cells were diluted tenfold in growth media, transferred to 1.5 mL Eppendorf tubes and incubated at 37 °C. At different time points between 15 min to 3 h, PI and Yo-Pro-1 (Sigma Aldrich) were added to final concentrations of 15 and 0.5 *μ*M, respectively and cells were kept on ice for 20 min before analysis. By incubating the cells on ice, the membrane re-sealing dynamics are slowed down,^21^ enhancing dye uptake by those cells that were permeable at the observation time points.

Fluorescence was measured using an LSR II flow cytometer (BD Biosciences, San Jose, CA). Both PI and Yo-Pro-1 were excited by a 488 nm laser and their emissions were collected by 695/40 and 530/40 band pass filters, respectively. Results were analyzed using the FACSDiVa v6.1 software (BD Biosciences). Gates were applied on forward and side scatter to exclude cell fragments and debris. A total of 10,000 counts inside the gating region were acquired and the measured fluorescence values were adjusted with a compensation factor to remove artifacts due to spectral overlap between the dyes. Finally, cells were classified as unstained, Yo-Pro-1 positive, PI positive or double stained. This classification was done by plotting fluorescence intensity in the two wavelengths of interest and defining four regions in the plot. These regions, as well as compensation factors, were adjusted by using positive and negative control samples. Positive controls were generated by exposure to ice-cold 70% ethanol for 30 min before staining with the desired dye.

### Expression of Caspase 3/7

At 4, 6 or 8 h after treatment, cells were detached using PBS with 0.5 mM EDTA and double stained to study the expression of Caspase 3/7 and cell membrane integrity. The time points evaluated were selected based on previous studies which showed a peak in Caspase activity around 6 h after the treatment.^31^,^50^ Caspase 3/7 expression was assessed using CellEvent™ Caspase-3/7 (Invitrogen) and membrane integrity was assessed using 4′,6-diamidino-2-phenylindole (DAPI, Panreac). Following the manufacturer’s instructions, 1 drop of CellEvent per mL of media was added and cells were incubated at 37 °C for 30 min. Afterwards, DAPI was added to a final concentration of 1 *μ*g/mL and cells were incubated at room temperature for 10 min prior to analysis.

Cells were analyzed by flow cytometry following a similar procedure as explained above. CellEvent was excited by a 488 nm laser and emission was collected by a 530/40 band pass filter. DAPI was excited by a 407 nm laser and emission was collected by a 460/50 band pass filter. No spectral overlap was detected between the two dyes. Positive control samples for Caspase 3/7 were generated by pharmacologically inducing apoptosis through treatment with 1 *μ*M of Staurosporine (Panreac). DAPI positive controls were prepared with ethanol treated cells as well as by delivering an electroporation treatment protocol in the presence of DAPI in the media. Positive and negative control samples were used to define regions and classify cells as unstained, Caspase expressing or DAPI positive.

## Results

### 3-h and 24-h Lesions in 3D Cell Cultures

**Figure 2:**
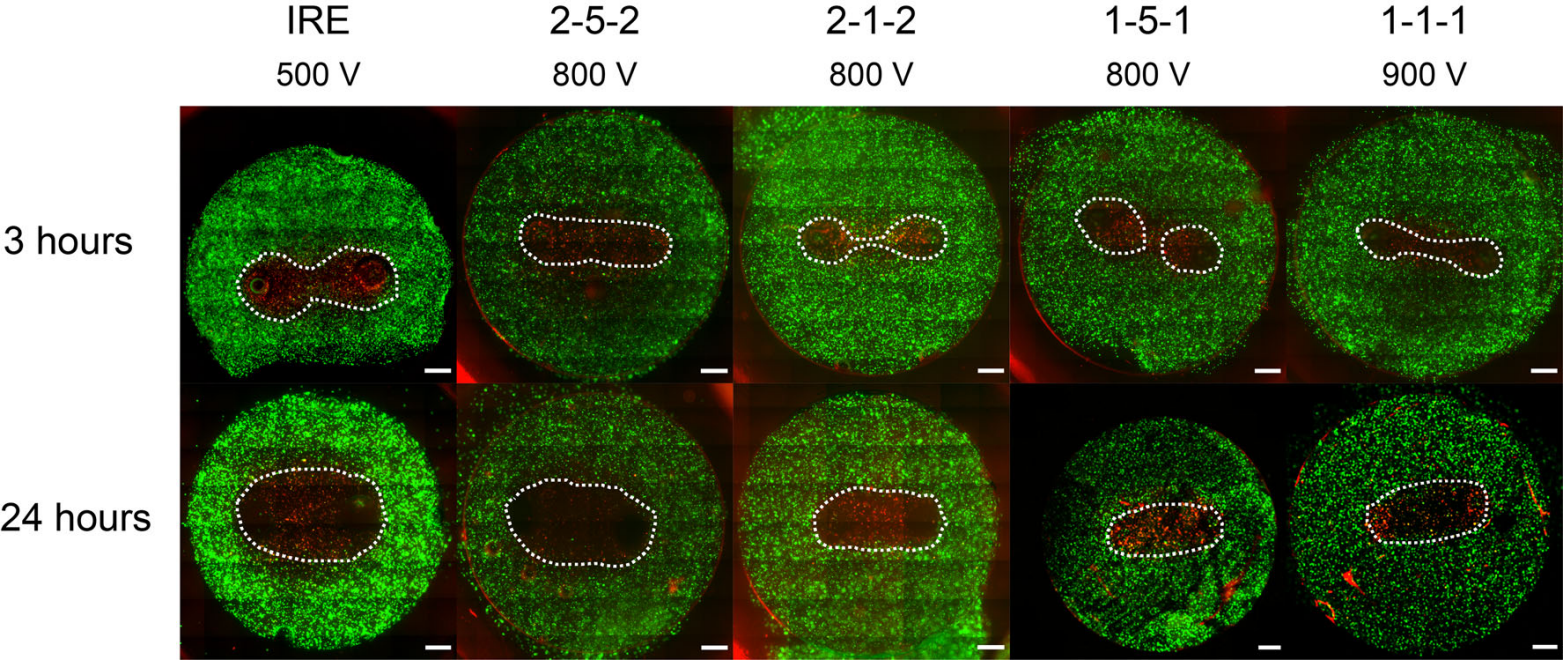
Twenty-four hour lesions are larger than 3 h lesions for all treatment protocols. Representative images of the lesions are shown for 3 and 24 h. Live cells display green fluorescence (Calcein AM) and dead cells display red fluorescence (PI) (Scale bars 1 mm).

**Figure 3:**
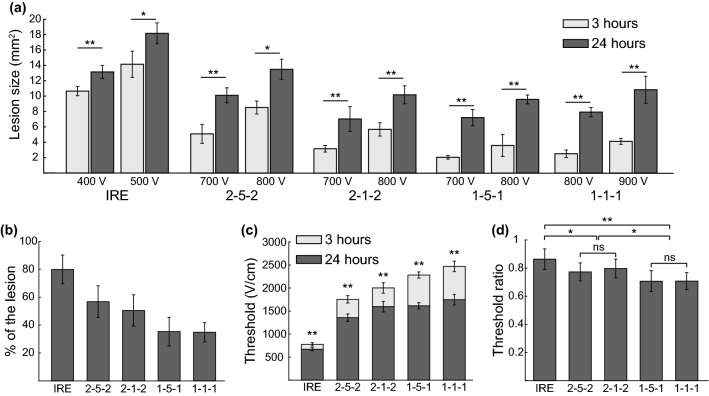
H-FIRE waveforms exhibit a pulse-length dependent increase in delayed cell death relative to IRE. (a) Lesion areas obtained at 3 and 24 h after treatment by using different treatment protocols (*n* ≥ 5 in all cases). (b) Average proportion of the lesion produced before 3 h. (c) Thresholds obtained at 3 and 24 h for each treatment protocol (*n* ≥ 10 in all cases). (d) Mean threshold ratios (threshold at 24 h divided by the threshold at 3 h). All results are presented as mean ± standard deviation (ns: *p *> 0.05, *p < 0.05, **p < 0.01).

An increase in lesion size was observed in treated 3D cell cultures between 3 and 24 h (Fig. 2). Statistically significant differences (*p *< 0.05) were found between the areas obtained at 3 and 24 h in all treatment protocols used in this study (Fig. 3a). The average fraction of the lesion that occurred after 3 h was larger in H-FIRE protocols than in the IRE protocol (Fig. 3b) and increased as pulse length was reduced. In the conventional IRE protocol, 80% of the total lesion occurred in less than 3 h. In the H-FIRE protocols with 1 *μ*s pulse length, this fraction was reduced to 35%. In addition, similar results were obtained for the H-FIRE protocols with the same pulse length regardless of the inter-pulse delay.

In every individual sample, the threshold was extracted from the measured lesion area by comparing it with the electric field distribution calculated numerically (see Fig. 1e). No statistical differences were found between thresholds obtained using different voltages for the same pulsing protocol and time point (*p *> 0.05, data not reported here). The average thresholds obtained at 3 and 24 h are presented in Fig. 3c and their values can be found in Table 1. Statistically significant differences were found between the thresholds at 3 and 24 h in all protocols. The range of electric fields that lead to delayed cell death (between 3 and 24 h) is larger in H-FIRE protocols than in the conventional IRE protocol and increases with decreasing pulse length. In contrast, the inter-pulse delay seems to have a minor effect on this range.

**Table 1:** Thresholds obtained in 3D cell cultures presented as mean ± standard deviation and expressed in V/cm. *p* values from Mann–Whitney–Wilcoxon test comparing the means at 3 and 24 h.

Protocol	3 h	24 h	*p* value
IRE	777 ± 46	671 ± 36	< 0.001
2-5-2	1754 ± 104	1356 ± 80	< 0.001
2-1-2	2002 ± 88	1596 ± 114	< 0.001
1-5-1	2283 ± 222	1614 ± 68	< 0.001
1-1-1	2468 ± 133	1746 ± 115	< 0.001

The ratios between the thresholds at 24 and 3 h are presented in Fig. 3d. The mean threshold ratio is lower in the H-FIRE protocols compared to the conventional IRE protocol and it decreases with the pulse length. Statistically significant differences were found between the conventional IRE protocol and all the H-FIRE protocols. When comparing the H-FIRE protocols, statistically significant differences were found between those protocols with different pulse length while no significant differences were found between the protocols with equal inter-pulse delay.

### Cell Death Dynamics in Cell Suspensions

**Figure 4:**
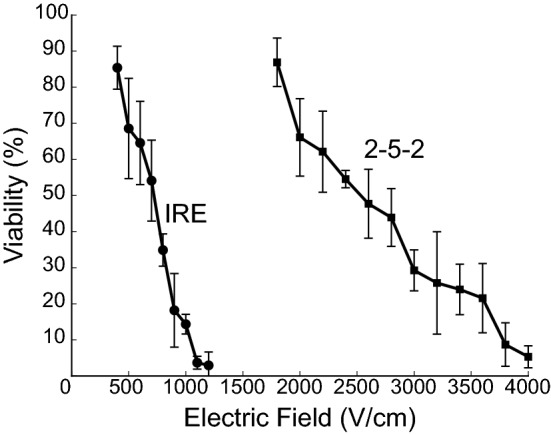
Cell viability after treating cell suspensions in cuvettes. Viability as a function of the electric field magnitude measured 24 h after cells in suspension were treated with conventional IRE and the 2-5-2 H-FIRE scheme. Results are presented as mean ± standard deviation (*n *≥ 3).

Cells in suspension were treated with the conventional IRE protocol and the 2-5-2 H-FIRE scheme. First, the 24 h thresholds for these protocols were measured. This was done by measuring cell viability 24 h after treatment for different electric field magnitudes (Fig. 4). Thresholds were extracted as the minimum electric field that produced a mean viability below 10%. A threshold of 1100 V/cm (110 V across 1 mm) was obtained for the conventional IRE protocol and a threshold of 3800 V/cm (380 V across 1 mm) was obtained for the 2-5-2 scheme. All of the following experiments were performed by applying the measured electric field thresholds.

**Figure 5:**
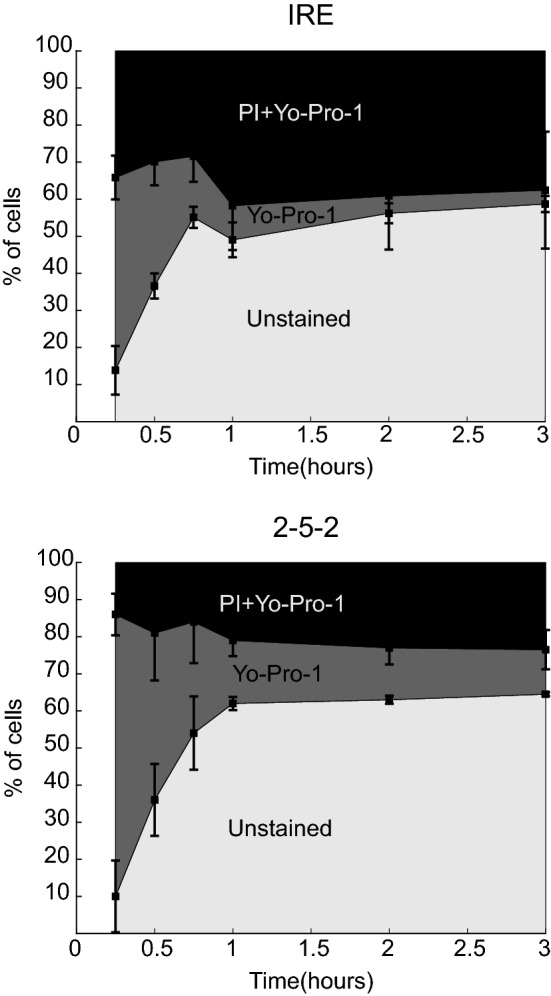
Time evolution of membrane permeability. Evolution of membrane permeability to PI and Yo-Pro-1 after treatment of cells in suspension with conventional IRE (100 *μ*s monopolar pulses) and 2-5-2 H-FIRE bursts with amplitudes of 1100 and 3800 V/cm, respectively. Squares show the mean percentage of cells permeable to PI and Yo-Pro-1 at different times after treatment (*n *≥ 4 for each time point). Error bars show the standard deviation.

Membrane permeability to PI and Yo-Pro-1 was assessed at different time points after treatment. Figure 5 shows that, in both treatments, after 15 min there is a large percentage of cells exhibiting permeability to Yo-Pro-1 but not to PI, especially in the H-FIRE samples. The percentage of PI permeable cells is larger in the samples treated with conventional IRE than in those treated with H-FIRE. Also, around 10% of the cells did not exhibit immediate uptake of either dye in both treatments. The percentage of PI permeable cells remains quite stable during all measured time points, especially after 1 h. In contrast, the percentage of Yo-Pro-1 permeable cells significantly decreases during the first hour and then remains stable in both cases. In samples treated with conventional IRE, this percentage is very low (< 4%) while in the H-FIRE samples it is around 15%.

**Figure 6:**
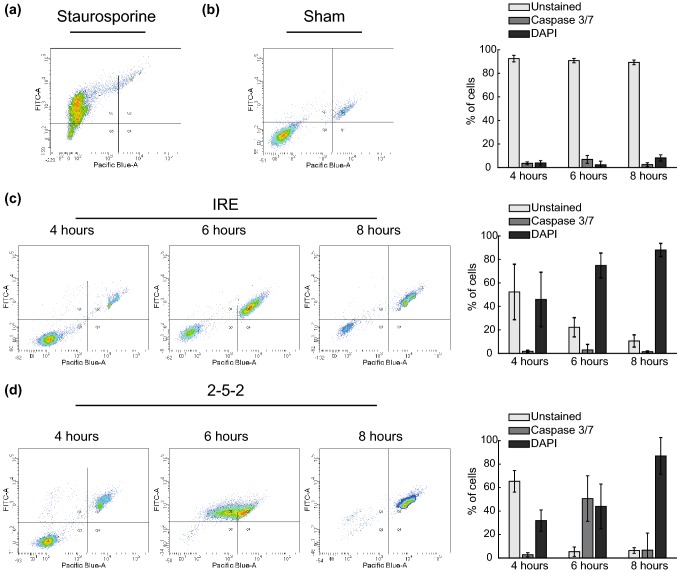
Cells treated with H-FIRE exhibit increased Caspase activity and a sharp loss of membrane integrity vs. IRE-treated cells, which gradually internalize DAPI without Caspase activation. Assessment of Caspase 3/7 activity and membrane integrity after treating cells in suspension with IRE pulses of 1100 V/cm and 2-5-2 bursts with 3800 V/cm of amplitude. Representative examples of the density plots obtained with flow cytometry analysis are presented together with the time evolution of the percentage of cells in the different populations. DAPI fluorescence was measured in the Pacific Blue channel and CellEvent Fluorescence in the FITC channel. Cells were classified as: Unstained (bottom left corner), Caspase 3/7 positive (upper left corner) and DAPI positive (upper right corner). The values in the bar plots are presented as mean ± standard deviation (*n *≥ 7 in all treatment groups and time points). (a) Density plot of cells treated with 1 *μ*M Staurosporine for 8 h (positive control for Caspase 3/7 expression) (b) Density plot and time evolution of the Sham group. (c) Density plots and time evolution of IRE treated cells. (d) Density plots and time evolution of cells treated with 2-5-2 H-FIRE scheme.

Finally, Caspase 3/7 activity and cell membrane integrity (DAPI internalization) were assessed at 4, 6 and 8 h after treatment. Results are presented in Fig. 6. In the H-FIRE treated samples, a peak in the percentage of cells expressing Caspase 3/7 is observed 6 h after treatment. Two hours later, most of the cells lost their membrane integrity. In contrast, the samples treated with conventional IRE gradually lost their membrane integrity between 4 and 8 h without displaying significant levels of Caspase 3/7 expression. Measurements at 5 and 7 h were also performed in IRE treated cells to further confirm the absence of Caspase 3/7 expression (results not presented here).

## Discussion

Our results show that conventional IRE and H-FIRE treatments can lead to either immediate or delayed cell death depending on the electric field magnitude. However, the range of electric fields that lead to delayed cell death is very narrow in the conventional IRE protocol, while in H-FIRE protocols this range is wider and can be increased by reducing the pulse length. This suggests that H-FIRE may offer greater control over the cell death mechanisms taking place after treatment. In other words, controlling the electric field exposure via parameters such as voltage, pulse length, and number of pulses may allow for selecting between immediate or delayed cell death mechanisms.

In a typical IRE treatment setup (two needle electrodes) the portion of the ablated volume undergoing immediate cell death would not depend on the absolute difference between electric field thresholds but on their relative difference. When comparing the electric fields that lead to immediate and delayed cell death in relative terms, the results show that the electric field ratios are lower in the H-FIRE protocols. Thus, a larger portion of the lesion can be comprised of cells dying through delayed mechanisms with H-FIRE compared to a conventional IRE protocol. In addition, this proportion can be increased by reducing the pulse length but does not depend on the inter-pulse delay.

The thresholds obtained for the H-FIRE protocols are between 2 and 4 times larger than those obtained for the conventional IRE protocol. Thus, higher voltages are required to treat the same volume with an H-FIRE protocol. However, a numerical study showed that the stimulation thresholds for nerve fibers are more than one order of magnitude larger in H-FIRE waveforms compared to 100 *μ*s monopolar pulses.^26^ The authors of that study attributed the divergent threshold dependences on the applied waveform to the geometrical differences between treated cells and nerve fibers which causes the latter to display significantly longer membrane charging times. As a consequence, despite requiring larger electric fields, H-FIRE protocols can largely reduce muscle contractions while maintaining treatment efficacy as shown *in vivo.*^2^

In both treatments, the evolution of PI permeability in Fig. 5 shows that even with electric fields around the threshold, there is a fraction of cells whose membrane integrity is not recovered, suggesting that a fraction of cells die almost immediately after the treatment regardless of the electric field. The fraction of DAPI positive cells at 4 h (Fig. 6) is very similar to the PI positive cells between 1 and 3 h. Therefore, those cells that recovered their membrane integrity during the first hour after the treatment remain viable at least until the 4 h time point. Finally, these remaining viable cells lose their membrane integrity between 4 and 8 h after the treatment. The observed time evolution is consistent with other experiments with conventional IRE pulses^32^ and nanosecond pulsed electric fields (nsPEFs).^31^ In addition, the results show the existence of two clearly identifiable cell death dynamics. First, a fraction of the cells cannot recover their membrane integrity and therefore suffer virtually instantaneous cell death (i.e. accidental cell death, ACD). Second, the rest of the cells recover their membrane integrity but eventually die, presumably through regulated cell death (RCD) mechanisms triggered by the treatment.

Yo-Pro-1 uptake can be either an early indicator of apoptosis^17^ or an indicator of membrane permeability.^4^ Yo-Pro-1 is smaller than PI which makes it a more sensitive marker of membrane permeability.^45^ In our experiments, a large fraction of Yo-Pro-1 positive cells was observed briefly after the treatments but significantly reduced within 1 h. This may be attributed to recovery of membrane integrity within this time interval. After 1 h, there is a small fraction of cells that remain permeable to Yo-Pro-1, which may be either an indicator of apoptosis or an indicator that these cells are unable to fully recover their membrane integrity.

Our results suggest that the mechanisms of delayed cell death are different between a typical IRE treatment and an H-FIRE scheme. In H-FIRE, this death is preceded by the expression of Caspase 3/7, but in conventional IRE, cells die through a pathway independent of Caspase 3/7. The activity of these proteins is widely used to classify cell death as it is one of the main hallmarks of apoptosis. Therefore, our results are consistent with an apoptotic cell death in the case of H-FIRE but not in the case of conventional IRE. However, further research is necessary to properly classify the death mechanisms in both cases.

The absence of Caspase 3/7 expression in cells treated with conventional IRE is consistent with recent studies that report RCD mechanisms independent of Caspase 3/7 expression such as pyroptosis or necroptosis.^25^,^55^ In the case of H-FIRE, histological examination of tissues found activation of Caspase 3,^29^,^43^ consistent with our results. However, another study showed evidence of pyroptotic and necroptotic cell death^35^ which would contradict this study. Nonetheless, it is important to note that we have only quantified Caspase 3/7 expression for a specific electric field magnitude. Thus, we cannot rule out the possibility that other RCD modes coexist within the range of electric fields that lead to delayed cell death.

In contrast with other waveforms, cell death mechanisms have been deeply studied after delivery of nsPEFs.^6^,^11^,^27^,^30^,^34^ From the numerous studies using nsPEFs, it can be deduced that cell death pathways depend on multiple variables such as cell type, pulse length, electric field strength and composition of the extracellular media.^5^ Thus, the results presented here regarding death mechanisms must be taken with caution as it is unclear how they can be extrapolated to other cell lines or pulse protocols.

Finally, it is worth discussing the implications of our results for *in vivo* tissue ablation. Dying cells release and expose at their surface molecules that act as signals to the immune system. These molecules depend on the cell death mechanism and can trigger inflammatory as well as immunogenic responses.^52^ While inflammation can lead to various complications, the adaptive response of the immune system can help prevent tumor recurrence and reduce metastases.^15^,^35^ Cells that suffer ACD release molecules that cause an inflammatory response, but seem to be limited in activating an adaptive immune response.^14^ Certain RCD mechanisms such as pyroptosis and necroptosis cause inflammation in a similar fashion, but they can also induce an adaptive immune response.^18^,^22^ Apoptosis, in contrast, has been classically regarded as a “silent” mode of cell death that does not cause inflammatory or immunogenic responses. However, it has been shown that, under certain circumstances, apoptosis can also trigger an immunogenic response. Interestingly, this has been observed after the delivery of nsPEFs.^28^ With our current knowledge it is not possible to provide a definite answer to the question of which cell death mode is the most desirable in any given ablation therapy. However, according to the literature, it seems that RCD mechanisms may be preferable over ACD. Studies have shown that both conventional IRE^8^,^16^ and H-FIRE^35^ treatments can trigger an anti-tumor immune response. Additionally, our results suggest that H-FIRE can reduce the inflammatory response compared to conventional IRE since the proportion of cells undergoing ACD is lower, but further research would be needed to evaluate this.

Our results also highlight the importance of choosing appropriate observation times to assess tissue ablation. In fact, previous studies already pointed out the importance of the observation times for histological evaluation of the ablations induced by conventional IRE protocols^46^,^47^ and showed that there is a large variability between different tissues.^46^ This may be even more relevant in H-FIRE treatments since the extent of delayed cell death is larger and therefore the risk of underestimating the lesion size is higher. In addition, in medical applications of IRE, tissue ablation is usually assessed by medical imaging techniques. Indeed, it has been shown that ultrasound^40^ and computed tomography^24^ imaging can accurately measure the lesion size within a few minutes after conventional IRE treatments. However, due to the larger proportion of delayed cell death in H-FIRE, immediate post-treatment imaging may not be adequate to assess the ablations. Thus, further research would be needed to investigate the optimal monitoring times after H-FIRE.

## Conclusions

We showed the existence of two clearly identifiable cell death dynamics consistent with accidental cell death (ACD) and regulated cell death (RCD) in cells treated with conventional IRE and H-FIRE. However, H-FIRE can largely reduce the fraction of cells that undergo ACD cell death compared to conventional IRE. In addition, H-FIRE may offer the possibility to select between ACD or RCD mechanisms with sufficient control over the electric fields delivered. Although the dynamics of delayed cell death are similar in both treatments, our results suggest that the RCD mechanisms leading to delayed cell death are different between IRE and H-FIRE.

## References

[1] Al-Sakere B, André F, Bernat C, Connault E, Opolon P, Davalos RV, Rubinsky B, Mir LM (2007). Tumor ablation with irreversible electroporation. PLoS ONE.

[2] Arena CB, Sano MB, Rossmeisl JH, Caldwell JL, Garcia PA, Rylander M, Davalos RV (2011). High-frequency irreversible electroporation (H-FIRE) for non-thermal ablation without muscle contraction. Biomed. Eng. Online.

[3] Arena CB, Szot CS, Garcia PA, Rylander MN, Davalos RV (2012). A three-dimensional in vitro tumor platform for modeling therapeutic irreversible electroporation. Biophys. J..

[4] Batista Napotnik T, Miklavčič D (2018). In vitro electroporation detection methods—an overview. Bioelectrochemistry.

[5] Beebe SJ, Miklavčič D (2017). Regulated and apoptotic cell death after nanosecond electroporation. Handbook of electroporation.

[6] Beebe SJ, Fox PM, Rec LJ, Somers K, Stark RH, Schoenbach KH (2002). Nanosecond pulsed electric field (nsPEF) effects on cells and tissues: apoptosis induction and tumor growth inhibition. IEEE Trans. Plasma Sci..

[7] Chai W, Zhang W, Wei Z, Xu Y, Shi J, Luo X, Zeng J, Cui M, Li J, Niu L (2017). Irreversible electroporation of the uterine cervix in a rabbit model. Biomed. Microdevices.

[8] Chen X, Ren Z, Yin S, Xu Y, Guo D, Xie H, Zhou L, Wu L, Jiang J, Li H, Sun J, Zheng S (2017). The local liver ablation with pulsed electric field stimulate systemic immune reaction against hepatocellular carcinoma (HCC) with time-dependent cytokine profile. Cytokine.

[9] Davalos RV, Bhonsle S, Neal RE (2015). Implications and considerations of thermal effects when applying irreversible electroporation tissue ablation therapy. Prostate.

[10] Edd JF, Horowitz L, Davalos RV, Mir LM, Rubinsky B (2006). In vivo results of a new focal tissue ablation technique: irreversible electroporation. IEEE Trans. Biomed. Eng..

[11] Ford WE, Ren W, Blackmore PF, Schoenbach KH, Beebe SJ (2010). Nanosecond pulsed electric fields stimulate apoptosis without release of pro-apoptotic factors from mitochondria in B16f10 melanoma. Arch. Biochem. Biophys..

[12] Fuchs Y, Steller H (2015). Live to die another way: modes of programmed cell death and the signals emanating from dying cells. Nat. Rev. Mol. Cell Biol..

[13] Galluzzi L (2018). Molecular mechanisms of cell death: recommendations of the Nomenclature Committee on Cell Death 2018. Cell Death Differ..

[14] Gamrekelashvili J, Ormandy LA, Heimesaat MM, Kirschning CJ, Manns MP, Korangy F, Greten TF (2012). Primary sterile necrotic cells fail to cross-prime CD8+ T cells. Oncoimmunology.

[15] Guo S, Jing Y, Burcus NI, Lassiter BP, Tanaz R, Heller R, Beebe SJ (2018). Nano-pulse stimulation induces potent immune responses, eradicating local breast cancer while reducing distant metastases. Int. J. Cancer.

[16] He C, Wang J, Sun S, Zhang Y, Li S (2019). Immunomodulatory effect after irreversible electroporation in patients with locally advanced pancreatic cancer. J. Oncol..

[17] Idziorek T, Estaquier J, De Bels F, Ameisen J-C (1995). YOPRO-1 permits cytofluorometric analysis of programmed cell death (apoptosis) without interfering with cell viability. J. Immunol. Methods.

[18] Inoue H, Tani K (2014). Multimodal immunogenic cancer cell death as a consequence of anticancer cytotoxic treatments. Cell Death Differ..

[19] Ivey JW, Latouche EL, Richards ML, Lesser GJ, Debinski W, Davalos RV, Verbridge SS (2017). Enhancing irreversible electroporation by manipulating cellular biophysics with a molecular adjuvant. Biophys. J..

[20] Kim HB, Sung CK, Baik KY, Moon KW, Kim HS, Yi JH, Jung JH, Moon MH, Choi OK (2013). Changes of apoptosis in tumor tissues with time after irreversible electroporation. Biochem. Biophys. Res. Commun..

[21] Kinosita KJ, Tsong TY (1977). Formation and resealing of pores of controlled sizes in human erythrocyte membrane. Nature.

[22] Krysko O, Aaes TL, Kagan VE, D’Herde K, Bachert C, Leybaert L, Vandenabeele P, Krysko DV (2017). Necroptotic cell death in anti-cancer therapy. Immunol. Rev..

[23] Lee EW, Loh CT, Kee ST (2007). Imaging guided percutaneous irreversible electroporation: ultrasound and immunohistological correlation. Technol. Cancer Res. Treat..

[24] Lee YJ, Lu DSK, Osuagwu F, Lassman C (2013). Irreversible electroporation in porcine liver: acute computed tomography appearance of ablation zone with histopathologic correlation. J. Comput. Assist. Tomogr..

[25] López-Alonso B, Hernáez A, Sarnago H, Naval A, Güemes A, Junquera C, Burdío JM, Castiella T, Monleón E, Gracia-Llanes J, Burdio F, Mejía E, Lucía O (2019). Histopathological and ultrastructural changes after electroporation in pig liver using parallel-plate electrodes and high-performance generator. Sci. Rep..

[26] Mercadal B, Arena CB, Davalos RV, Ivorra A (2017). Avoiding nerve stimulation in irreversible electroporation: a numerical modeling study. Phys. Med. Biol..

[27] Napotnik TB, Wu Y-H, Gundersen MA, Miklavčič D, Vernier PT (2012). Nanosecond electric pulses cause mitochondrial membrane permeabilization in Jurkat cells. Bioelectromagnetics.

[28] Nuccitelli R, McDaniel A, Anand S, Cha J, Mallon Z, Berridge JC, Uecker D (2017). Nano-pulse stimulation is a physical modality that can trigger immunogenic tumor cell death. J. Immunother. Cancer.

[29] O’Brien TJ, Passeri M, Lorenzo MF, Sulzer JK, Lyman WB, Swet JH, Vrochides D, Baker EH, Iannitti DA, Davalos RV, McKillop IH (2019). Experimental high-frequency irreversible electroporation using a single-needle delivery approach for nonthermal pancreatic ablation in vivo. J. Vasc. Interv. Radiol..

[30] Pakhomov AG, Phinney A, Ashmore J, Walker K, Kolb JF, Kono S, Schoenbach KH, Murphy MR (2004). Characterization of the cytotoxic effect of high-intensity, 10-ns duration electrical pulses. IEEE Trans. Plasma Sci..

[31] Pakhomova ON, Gregory BW, Semenov I, Pakhomov AG (2013). Two modes of cell death caused by exposure to nanosecond pulsed electric field. PLoS ONE.

[32] Qin Q, Xiong ZA, Liu Y, Yao CG, Zhou W, Hua YY, Wang ZL (2016). Effects of irreversible electroporation on cervical cancer cell lines in vitro. Mol. Med. Rep..

[33] R Core Team. R: A Language and Environment for Statistical Computing, 2014.

[34] Ren W, Sain NM, Beebe SJ (2012). Nanosecond pulsed electric fields (nsPEFs) activate intrinsic caspase-dependent and caspase-independent cell death in Jurkat cells. Biochem. Biophys. Res. Commun..

[35] Ringel-Scaia VM, Beitel-White N, Lorenzo MF, Brock RM, Huie KE, Coutermarsh-Ott S, Eden K, McDaniel DK, Verbridge SS, Rossmeisl JH, Oestreich KJ, Davalos RV, Allen IC (2019). High-frequency irreversible electroporation is an effective tumor ablation strategy that induces immunologic cell death and promotes systemic anti-tumor immunity. EBioMedicine.

[36] Rubinsky B, Onik G, Mikus P (2007). Irreversible electroporation: a new ablation modality—clinical implications. Technol. Cancer Res. Treat..

[37] Sachet M, Liang YY, Oehler R (2017). The immune response to secondary necrotic cells. Apoptosis.

[38] Sano MB, Arena CB, Bittleman KR, DeWitt MR, Cho HJ, Szot CS, Saur D, Cissell JM, Robertson J, Lee YW, Davalos RV (2015). Bursts of bipolar microsecond pulses inhibit tumor growth. Sci. Rep..

[39] Sano MB, Arena CB, DeWitt MR, Saur D, Davalos RV (2014). In-vitro bipolar nano- and microsecond electro-pulse bursts for irreversible electroporation therapies. Bioelectrochemistry.

[40] Schmidt CR, Shires P, Mootoo M (2012). Real-time ultrasound imaging of irreversible electroporation in a porcine liver model adequately characterizes the zone of cellular necrosis. Hpb.

[41] Schneider CA, Rasband WS, Eliceiri KW (2012). NIH Image to ImageJ: 25 years of image analysis. Nat. Methods.

[42] Siddiqui IA, Kirks RC, Latouche EL, DeWitt MR, Swet JH, Baker EH, Vrochides D, Iannitti DA, Davalos RV, McKillop IH (2017). High-frequency irreversible electroporation: safety and efficacy of next-generation irreversible electroporation adjacent to critical hepatic structures. Surg. Innov..

[43] Siddiqui IA, Latouche EL, DeWitt MR, Swet JH, Kirks RC, Baker EH, Iannitti DA, Vrochides D, Davalos RV, McKillop IH (2016). Induction of rapid, reproducible hepatic ablations using next-generation, high frequency irreversible electroporation (H-FIRE) in vivo. HPB.

[44] Szot CS, Buchanan CF, Freeman JW, Rylander MN (2011). 3D in vitro bioengineered tumors based on collagen I hydrogels. Biomaterials.

[45] Vernier PT, Sun Y, Gundersen MA (2006). Nanoelectropulse-driven membrane perturbation and small molecule permeabilization. BMC Cell Biol..

[46] Vogel JA, Van Veldhuisen E, Agnass P, Crezee J, Dijk F, Verheij J, Van Gulik TM, Meijerink MR, Vroomen LG, Van Lienden KP, Besselink MG (2016). Time-dependent impact of irreversible electroporation on pancreas, liver, blood vessels and nerves: a systematic review of experimental studies. PLoS ONE.

[47] Vogel JA, van Veldhuisen E, Alles LK, Busch OR, Dijk F, van Gulik TM, Huijzer GM, Besselink MG, van Lienden KP, Verheij J (2019). Time-dependent impact of irreversible electroporation on pathology and ablation size in the porcine liver: a 24-hour experimental study. Technol. Cancer Res. Treat..

[48] Wasson EM, Ivey JW, Verbridge SS, Davalos RV (2017). The feasibility of enhancing susceptibility of glioblastoma cells to IRE using a calcium adjuvant. Ann. Biomed. Eng..

[49] Weaver JC, Smith KC, Esser AT, Son RS, Gowrishankar TR (2012). Bioelectrochemistry A brief overview of electroporation pulse strength—duration space: a region where additional intracellular effects are expected. Bioelectrochemistry.

[50] Xiong Z, Yao C, Zhou W, Liu Y, Li C (2012). Low voltage irreversible electroporation induced apoptosis in HeLa cells. J. Cancer Res. Ther..

[51] Yao C, Dong S, Zhao Y, Lv Y, Liu H, Gong L, Ma J, Wang H, Sun Y (2017). Bipolar microsecond pulses and insulated needle electrodes for reducing muscle contractions during irreversible electroporation. IEEE Trans. Biomed. Eng..

[52] Yatim N, Cullen S, Albert ML (2017). Dying cells actively regulate adaptive immune responses. Nat. Rev. Immunol..

[53] Zhang W, Chai W, Zeng J, Chen J, Bi L, Niu L (2017). Irreversible electroporation for the treatment of rabbit VX2 breast cancer. Biomed. Microdevices.

[54] Zhang Z, Li W, Procissi D, Tyler P, Omary RA, Larson AC (2014). Rapid dramatic alterations to the tumor microstructure in pancreatic cancer following irreversible electroporation ablation. Nanomedicine.

[55] Zhang Y, Lyu C, Liu Y, Lv Y, Chang TT, Rubinsky B (2018). Molecular and histological study on the effects of non-thermal irreversible electroporation on the liver. Biochem. Biophys. Res. Commun..

